# Supplementary figures and images for: Plasma trimethylamine N-oxide and its metabolic precursors and risk of mortality, cardiovascular and renal disease in individuals with type 2-diabetes and albuminuria
Source: PLoS One. 2021 Mar 3;16(3):e0244402. doi: 10.1371/journal.pone.0244402 (PMC7928450; doi:10.1371/journal.pone.0244402)

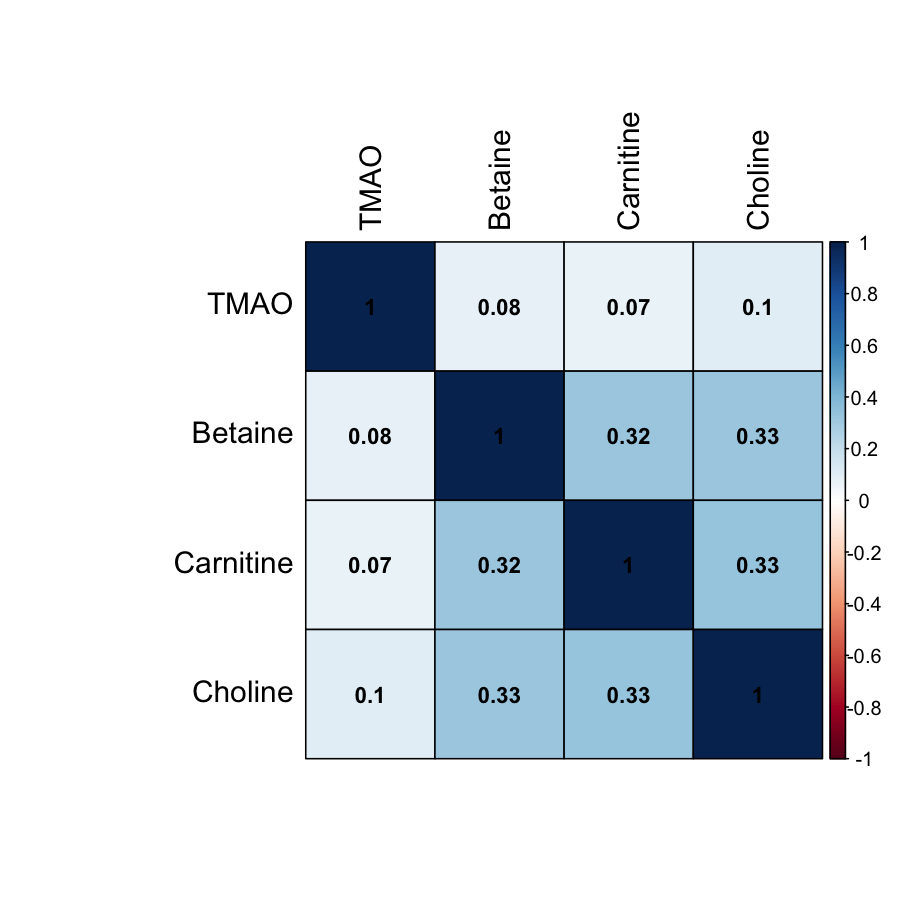

Supplement: S1 Fig — Numbers are R2 and all p-values <0.0001. TMAO = trimethylamine N-oxide. (TIFF) [file pone.0244402.s001.tiff]
